# Diagnostic Potential and Interactive Dynamics of the Colorectal Cancer Virome

**DOI:** 10.1128/mBio.02248-18

**Published:** 2018-11-20

**Authors:** Geoffrey D. Hannigan, Melissa B. Duhaime, Mack T. Ruffin, Charlie C. Koumpouras, Patrick D. Schloss

**Affiliations:** a Department of Microbiology and Immunology, University of Michigan, Ann Arbor, Michigan, USA; b Department of Ecology and Evolutionary Biology, University of Michigan, Ann Arbor, Michigan, USA; c Department of Family and Community Medicine, Pennsylvania State University Hershey Medical Center, Hershey, Pennsylvania, USA; UCLA School of Medicine

**Keywords:** bacteriophage, colorectal cancer, diagnostic, microbial ecology, microbiome, microbiota, random forest, virome

## Abstract

Human viruses (those that infect human cells) have been associated with many cancers, largely due to their mutagenic and functionally manipulative abilities. Despite this, cancer microbiome studies have focused almost exclusively on bacteria instead of viruses. We began evaluating the cancer virome by focusing on colorectal cancer, a primary cause of morbidity and mortality throughout the world and a cancer linked to altered colonic bacterial community compositions but with an unknown association with the gut virome. We used 16S rRNA gene, whole shotgun metagenomic, and purified virus metagenomic sequencing of stool to evaluate the differences in human colorectal cancer virus and bacterial community composition. Through random forest modeling, we identified differences in the healthy and colorectal cancer viromes. The cancer-associated virome consisted primarily of temperate bacteriophages that were also predicted to be bacterium-virus community network hubs. These results provide foundational evidence that bacteriophage communities are associated with colorectal cancer and potentially impact cancer progression by altering the bacterial host communities.

## Abstract (precis)

Colorectal cancer is a leading cause of cancer-related death in the United States and worldwide. Its risk and severity have been linked to colonic bacterial community composition. Although human-specific viruses have been linked to other cancers and diseases, little is known about colorectal cancer virus communities. We addressed this knowledge gap by identifying differences in colonic virus communities in the stool of colorectal cancer patients and how they compared to bacterial community differences. The results suggested an indirect role for the virome in impacting colorectal cancer by modulating the associated bacterial community. These findings both support the idea of a biological role for viruses in colorectal cancer and provide a new understanding of basic colorectal cancer etiology.

## INTRODUCTION

The human gut virome is the community of all viruses found in the gut, including bacteriophages (viruses that infect only bacteria), eukaryotic viruses (viruses that infect only eukaryotic cells), and human-specific viruses (viruses that infect only human cells). Due to their mutagenic abilities and propensity for functional manipulation, human viruses are strongly associated with and in many cases cause cancer (1–4). Because bacteriophages are crucial for bacterial community stability and composition (5–7) and because members of those bacterial communities have been implicated as oncogenic agents (8–11), bacteriophages have the potential to indirectly impact cancer as well. The gut virome, therefore, has the potential to be associated with and, potentially, to impact human cancer. Altered human virome composition and diversity have already been identified in various diseases, including periodontal disease (12), HIV (13), cystic fibrosis (14), disease resulting from antibiotic exposure (15, 16), urinary tract infections (17), and inflammatory bowel disease (18). The strong association of bacterial communities with colorectal cancer (CRC), the previous identification of human-specific viruses that cause cancer, and the precedent for the virome to impact other human diseases suggest that colorectal cancer may be associated with altered virus communities.

Colorectal cancer is the second leading cause of cancer-related deaths in the United States (19). The U.S. National Cancer Institute estimates that over 1.5 million Americans were diagnosed with colorectal cancer in 2016 and that over 500,000 Americans died from the disease (19). Growing evidence suggests that an important component of colorectal cancer etiology may be perturbations in the colonic bacterial community (8, 10, 11, 20, 21). Work in this area has led to a proposed disease model in which bacteria colonize the colon, develop biofilms, promote inflammation, and enter an oncogenic synergy with the cancerous human cells (22). This association also has allowed researchers to leverage bacterial community signatures as biomarkers to enable accurate, noninvasive colorectal cancer detection from stool (8, 23, 24). While an understanding of colorectal cancer bacterial communities has proven fruitful both for disease classification and for identification of the underlying disease etiology, bacteria represent only a subset of the colon microbiome. Viruses are another important component of the colon microbial community and have yet to be studied in the context of colorectal cancer. We evaluated disruptions in virus and bacterial community composition in a human cohort whose stool was sampled at the three relevant stages of cancer development: healthy, adenomatous, and cancerous.

Colorectal cancer progresses in a stepwise process that begins when healthy tissue develops into a precancerous polyp (i.e., adenoma) in the large intestine (25). If not removed, the adenoma may develop into a cancerous lesion that can invade and metastasize, leading to severe illness and death. Progression to cancer can be prevented when precancerous adenomas are detected and removed during routine screening (26, 27). Survival for colorectal cancer patients may exceed 90% when the lesions are detected early and removed (26). Thus, work that aims to facilitate early detection and prevention of progression beyond early cancer stages has great potential to inform therapeutic development.

Here we begin to address the knowledge gap with respect to whether virus community composition is altered in colorectal cancer and, if it is, how such differences might impact cancer progression and severity. We also aimed to evaluate the virome’s potential for use as a diagnostic biomarker. The implications of this study are 3-fold. First, this work supports the idea of a biological role for the virome in colorectal cancer development and suggests that more than the bacterial members of the associated microbial communities are involved in the process. Second, we present a supplementary virus-based approach for classification modeling of colorectal cancer using stool samples. Third, we provide initial support for the idea of the importance of studying the virome as a component of the microbiome ecological network, especially in cancer.

## RESULTS

### Sample collection and processing.

**FIG 1:**
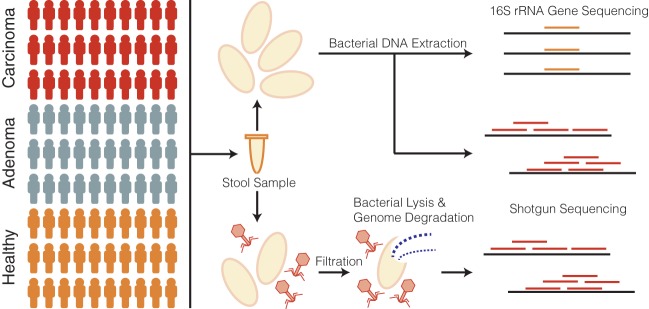
Cohort and sample processing outline. Thirty subject stool samples were collected from healthy subjects and adenoma (precancer) and carcinoma (cancer) patients. Stool samples were split into two aliquots, the first of which was used for bacterial sequencing and the second of which was used for virus sequencing. Bacterial sequencing was done using both 16S rRNA amplicon and whole-metagenomic shotgun sequencing techniques. Virus samples were purified for viruses using filtration and a combination of chloroform (bacterial lysis) and DNase (exposed genomic DNA degradation). The resulting encapsulated virus DNA was sequenced using whole-metagenomic shotgun sequencing.

Our study cohort consisted of stool samples collected from 90 human subjects, 30 of whom had healthy colons, 30 of whom had adenomas, and 30 of whom had carcinomas (Fig. 1). Half of each stool sample was used to sequence the bacterial communities using both 16S rRNA gene and shotgun sequencing techniques. The 16S rRNA gene sequencing was performed for a previous study, and the sequences were reanalyzed using contemporary methods (8). The other half of each stool sample was purified for virus-like particles (VLPs) before genomic DNA extraction and shotgun metagenomic sequencing were performed. In the VLP purification, cells were disrupted and extracellular DNA degraded (Fig. 1) to allow the exclusive analysis of viral DNA within virus capsids. In this manner, the extracellular virome of encapsulated viruses was targeted.

Each extraction was performed with a blank buffer control to detect contaminants from reagents or other unintentional sources. Only one of the nine controls contained detectable DNA at a minimal concentration of 0.011 ng/µl, thus providing evidence of the enrichment and purification of VLP genomic DNA over potential contaminants (see Fig. S1A in the supplemental material). As expected, these controls yielded few sequences and were almost entirely removed in rarefying the data sets to a common number of sequences (Fig. S1B). The high-quality phage and bacterial sequences were assembled into highly covered contigs longer than 1 kb (Fig. S2). Because contigs represent genome fragments, we further clustered related bacterial contigs into operational genomic units (OGUs) and viral contigs into operational viral units (OVUs) (Fig. S2 and S3) to approximate organismal units.

10.1128/mBio.02248-18.1 FIG S1 Basic quality control metrics. (A) VLP genomic DNA yield from all sequenced samples. Each bar represents a sample; the samples are grouped and colored by their associated disease group or as no-DNA negative controls. (B) Sequence yield following quality control, including quality score filtering and human decontamination. Borders consisting of dashed lines indicate a rarefaction level (10^6^ reads) in which all samples with lower sequence yields below than that level were excluded from downstream analysis. After rarefaction and removal of samples with fewer than 10^6^ reads, 27 healthy, 28 cancerous, 27 adenomatous, and 3 negative-control samples remained. Download FIG S1, EPS file, 0.02 MB. Copyright © 2018 Hannigan et al. 2018 Hannigan et al. This content is distributed under the terms of the Creative Commons Attribution 4.0 International license.

10.1128/mBio.02248-18.2 FIG S2 Length and coverage statistics. (A) Heated scatter plot demonstrating the distribution of contig coverage (number of sequences mapping to each contig) and contig length data for the virus metagenomic sample set. (B) Scatter plot illustrating the distribution of operational viral unit (OVU) length and sequence coverage data for the virus metagenomic sample set. (C) Heated scatter plot demonstrating the distribution of contig coverage and length data for the whole-metagenomic sample set. (D) Scatter plot illustrating the distribution of operational genomic unit (OGU) length and sequence coverage data for the whole-metagenomic sample set. Download FIG S2, EPS file, 0.9 MB. Copyright © 2018 Hannigan et al. 2018 Hannigan et al. This content is distributed under the terms of the Creative Commons Attribution 4.0 International license.

10.1128/mBio.02248-18.3 FIG S3 Operational genomic unit composition statistics. (A) Strip chart demonstrating the length and frequency of contigs within each operational genomic unit of the virome sample set. The *y* axis is the operational genomic unit identifier, and *x* axis is the length of each contig; each dot represents a contig found within the specified operational genomic unit. (B) Density plot (analogous to histogram) of the number of virome operational genomic units containing the specific number of contigs, as indicated by the *x* axis. (C and D) Sample plots constructed as described for panels C and D but for the whole-metagenomic sample set. Download FIG S3, TIF file, 1.5 MB. Copyright © 2018 Hannigan et al. 2018 Hannigan et al. This content is distributed under the terms of the Creative Commons Attribution 4.0 International license.

### Unaltered diversity in colorectal cancer.

Microbiome and disease associations are often described as being of an altered diversity (i.e., "dysbiotic"). Therefore, we first evaluated the influence of colorectal cancer on virome OVU diversity. We evaluated differences in communities between disease states using the Shannon diversity, richness, and Bray-Curtis metrics. We observed no significant alterations in either Shannon diversity or richness in the diseased states compared to the healthy state (Fig. S4C and D). There was no statistically significant clustering of the disease groups (analysis of similarity [ANOSIM] *P* value = 0.6) (Fig. S4). Notably, there were significant differences between the few blank controls that remained after rarefaction of the data and the other study groups (ANOSIM *P* value < 0.001) (Fig. S5), further supporting the quality of the sample set. In summary, standard alpha and beta diversity metrics were insufficient for capturing virus community differences between disease states (Fig. S4). This is consistent with what had been observed when the same metrics were applied to 16S rRNA gene sequences and metagenomic samples (8, 23, 24) and points to the need for alternative approaches to detect the impact of colorectal cancer disease state on these community structures.

10.1128/mBio.02248-18.4 FIG S4 Diversity calculations comparing cancer states of the colorectal virome, based on relative abundances of operational genomic units in each sample. (A) Nonmetric multidimensional scaling (NMDS) ordination of community samples, colored for cancerous (green), precancerous (red), and healthy (yellow) samples. (B) Differences in means between disease group centroids with 95% confidence intervals based on an ANOSIM with a *post hoc* multivariate Tukey test. Results of comparisons (indicated on the *y* axis) in which the intervals crossed the zero mean difference line (dashed line) were not significantly different. (C and D) Shannon diversity (C) and richness alpha diversity (D) quantification, comparing precancerous (gray), cancerous (red), and healthy (tan) states. Download FIG S4, EPS file, 0.1 MB. Copyright © 2018 Hannigan et al. 2018 Hannigan et al. This content is distributed under the terms of the Creative Commons Attribution 4.0 International license.

10.1128/mBio.02248-18.5 FIG S5 Beta-diversity analysis comparing Bray-Curtis dissimilarity data between disease state and negative-control community structures that were captured following sequence rarefaction. Data represent differences in means between disease group centroids with 95% confidence intervals based on an ANOSIM with a *post hoc* multivariate Tukey test. Results of comparisons in which the intervals crossed the zero mean difference line (dashed line) were not significantly different. Download FIG S5, EPS file, 0.02 MB. Copyright © 2018 Hannigan et al. 2018 Hannigan et al. This content is distributed under the terms of the Creative Commons Attribution 4.0 International license.

### Virome composition in colorectal cancer.

In contrast to the diversity metrics discussed above, OTU-based relative abundance profiles generated from 16S rRNA gene sequences are effective for classifying stool samples as originating from individuals with healthy, adenomatous, or cancerous colons (8, 23). By using classification models instead of attempting to identify individual differentially abundant OTUs, those and other studies have been successful in capturing complex community relationships in which differences in taxonomic relative abundances are considered in the context of other taxa. The exceptional performance of bacteria in these classification models supports the idea of a role for bacterial functionality in colorectal cancer. We built on these findings by evaluating the ability of virus community signatures to classify stool samples and compared their performance to that of models built using bacterial community signatures.

**FIG 2:**
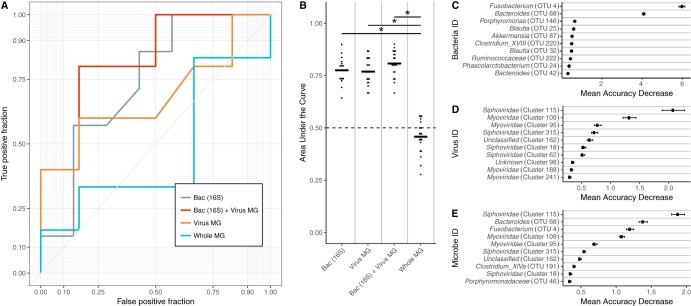
Results from healthy versus cancer classification models built using virome signatures, bacterial 16S rRNA gene sequence signatures, whole-metagenomic signatures, and a combination of virome and 16S rRNA gene sequence signatures. (A) An example receiver operating characteristic (ROC) curve for visualizing the performance of each of the models for classifying stool as coming from either an individual with a cancerous colon or an individual with a healthy colon. (B) Quantification of the AUC variation for each model and how it compared to each of the other models based on 15 iterations. A pairwise Wilcoxon test with a false-discovery-rate multiple-hypothesis correction demonstrated that all models are significantly different from each other (*P* value < 0.01). (C) Mean decrease in accuracy (measurement of importance) of each operational taxonomic unit within the 16S rRNA gene classification model when removed from the classification model. The mean is represented by a point, and bars represent standard errors. ID, identifier. (D) Mean decrease in accuracy of each operational virus unit in the virome classification model. (E) Mean decrease in accuracy of each operational genomic unit and operational taxonomic unit in the model using both 16S rRNA gene and virome features.

To identify the altered virus communities associated with colorectal cancer, we built and tested random forest models for classifying stool samples as belonging to individuals with either cancerous or healthy colons. We confirmed that our bacterial 16S rRNA gene model replicated the performance of the original report, which used logit models instead of random forest models (Fig. 2A) (8). We then compared the bacterial OTU model to a model built using OVU relative abundances. The viral model performed as well as the bacterial model (corrected *P* value = 0.4), with the viral and bacterial models achieving mean area under the curve (AUC) values of 0.792 and 0.809, respectively (Fig. 2A and B). To evaluate the ability of both bacterial and viral biomarkers to classify samples, we built a combined model that used both bacterial and viral community data. The combined model did not yield a statistically significant performance improvement beyond the performance of the viral (corrected *P* value = 0.4) and bacterial (corrected *P* value = 0.08) models, yielding an AUC of 0.768 (Fig. 2A and B).

We compared viral metagenomic methods to bacterial metagenomic methods by building a viral model and a model using OGU relative abundance profiles from bacterial metagenomic shotgun sequencing data. This bacterial model performed worse than the other models (mean AUC = 0.474) (Fig. 2A and B). To determine the cause of the discrepancy between the two bacterial sequencing methods, we attempted to compare the approaches at a common sequencing depth. This revealed that the bacterial 16S rRNA gene model was strongly driven by sparse and low-abundance OTUs (Fig. S6). Removal of OTUs with a median abundance of zero resulted in the removal of six OTUs and in a loss of model performance comparable to what was observed in the metagenome-based model (Fig. S6A). The majority of these OTUs had a relative abundance of lower than 1% across the samples (Fig. S6B). Although the features in the viral model also were of low abundance (Fig. S8F), the coverage was sufficient for high model performance, likely because viral genomes are orders of magnitude smaller than bacterial genomes.

10.1128/mBio.02248-18.6 FIG S6 Comparison of bacterial 16S rRNA classification models with and without OTUs whose median relative abundances are greater than zero. (A) Classification model performance (measured as area under the curve) for bacteria models using 16S rRNA data both with and without filtering of samples whose medians were zero. Significance was calculated using a Wilcoxon rank sum test, and the resulting *P* value is shown. The random area under the curve (0.5) is marked with a dashed line. (B) Relative abundances of the six bacterial OTUs removed by filtration for OTUs with a median relative abundance of zero. OTU relative abundance data are separated for groups of healthy (red) and cancerous (gray) samples. Data corresponding to a relative abundance of 1% are marked by the dashed line. Download FIG S6, EPS file, 0.1 MB. Copyright © 2018 Hannigan et al. 2018 Hannigan et al. This content is distributed under the terms of the Creative Commons Attribution 4.0 International license.

The association between the bacterial and viral communities and colorectal cancer was driven by a few important microbes. *Fusobacterium* was the primary driver of the bacterial association with colorectal cancer, which is consistent with its previously described oncogenic potential (Fig. 2C) (22). The virome signature also was driven by a few OVUs, suggesting a role for these viruses in tumorigenesis (Fig. 2D). Note that while these viruses were driving the signature, the magnitude of their importance and the significance of those values were noticeably less than those corresponding to the bacterial 16S signature, suggesting that, unlike what is observed in the bacteria, there are many viruses that are associated with the cancerous state. The identified viruses were bacteriophages, belonging to *Siphoviridae* and *Myoviridae* and to phage taxa that could not be confidently identified beyond their broad phage identification (i.e., "unclassified"). Many of the important viruses that were confirmed to not have genomic similarity to known bacterial genomes were unidentifiable (denoted "unknown"). This is common in viromes across habitats; studies have reported that as much as 95% of virus sequences belong to unknown genomic units (14, 28–30). When the bacterial and viral community signatures were combined, both bacterial and viral organisms were found to drive the community association with cancer (Fig. 2E).

### Phage influence between CRC stages.

Because previous work has identified shifts in which bacteria were most important at different stages of colorectal cancer (8, 20, 22), we explored whether shifts in the relative influences of phages could be detected between healthy, adenomatous, and cancerous colons. We evaluated community shifts between the disease stage transitions (healthy to adenomatous and adenomatous to cancerous) by building random forest models to compare only the diagnosis groups present around the transitions. While bacterial OTU models performed equally well for all disease class comparisons, the virome model performances differed (Fig. S7A and B). Like bacteria (Fig. S7F to H), different virome members were important between the healthy-to-adenomatous and adenomatous-to-cancerous stages (Fig. S7C to E).

10.1128/mBio.02248-18.7 FIG S7 Transition of colorectal cancer importance through disease progression. (A and B) Virus (A) and 16S rRNA gene model (B) performance (AUC) determined on the basis of discrimination of all binary combinations of disease types. The blue line represents data corresponding to the mean performance determined from multiple random iterations. (C to E) Top 10 important phage OVUs, classifying each combination of disease state, as measured by the mean decrease in the accuracy metric. The mean is represented by a point, and bars represent standard errors. Disease comparisons are specified in the top left corner of each panel. (F to H) Top 10 important bacterial 16S rRNA gene OTUs, classifying each disease state combination. Download FIG S7, EPS file, 0.1 MB. Copyright © 2018 Hannigan et al. 2018 Hannigan et al. This content is distributed under the terms of the Creative Commons Attribution 4.0 International license.

After evaluating our ability to classify samples between two disease states, we employed a three-class random forest model that included all disease states. The 16S rRNA gene model yielded a mean AUC of 0.765 and outperformed the viral community model, which yielded a mean AUC of 0.658 (*P* value < 0.001) (Fig. S8A to C). The microbes important for the healthy versus cancer and healthy versus adenoma models were also important for the three-class model (Fig. S8D and E). The most important bacterium in the two-class and three-class models was the same *Fusobacterium* sp. (OTU 4) (Fig. 2C; see also Fig. S8D). The most important viruses in the three-class model were identified as bacteriophages (Fig. 2; see also Fig. S8E), but not all of the important OVUs were of increased abundance in the diseased state (Fig. S8F).

10.1128/mBio.02248-18.8 FIG S8 (A and B) ROC curves from virome (A) and bacterial (B) 16S three-class random forest models tuned using mean AUC. Each curve represents the ability of the specified class to be classified against the other two classes. (C) Quantification of the mean AUC variation for each model based on 10 model iterations. A pairwise Wilcoxon test with a Bonferroni multiple-hypothesis correction demonstrated that the models are significantly different (alpha = 0.01). (D and E) Mean decrease in accuracy after removal of virome operational genomic units (D) and bacterial 16S OTUs (E) from the respective three-class classification models. Data are based on 25 iterations. (F) Relative abundances of the six most important virome OVUs in the model, with the most important on the right. The line indicates abundance means. Download FIG S8, EPS file, 0.1 MB. Copyright © 2018 Hannigan et al. 2018 Hannigan et al. This content is distributed under the terms of the Creative Commons Attribution 4.0 International license.

### Phage dominance in CRC virome.

Differences in the colorectal cancer virome could have been driven by eukaryotic (human) viruses or by bacteriophages. To better understand the types of viruses that were important for colorectal cancer, we identified the virome OVUs as being similar to either eukaryotic viruses or bacteriophages. The most important viruses in the classification model were identified as bacteriophages (Fig. S8). Overall, we were able to identify 78.8% of the OVUs as known viruses, and 93.8% of those viral OVUs aligned to bacteriophage reference genomes. Note that this could have been influenced by our methodological biases against enveloped viruses (more common among eukaryotic viruses than bacteriophage), due to the use of chloroform and DNase treatment for purification.

**FIG 3:**
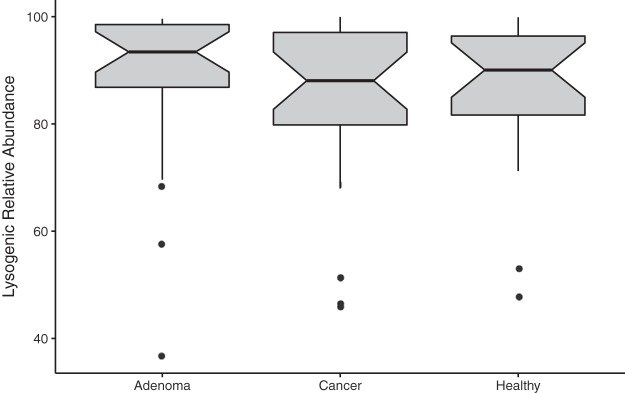
Lysogenic phage relative abundance in disease states. Phage OVUs were predicted to be either lytic or lysogenic, and the relative abundances of lysogenic phages were quantified and are represented as a box plot. None of the data from the disease groups were statistically significant.

We evaluated whether the phages in the community were primarily lytic (i.e., obligately lysed their hosts after replication) or temperate (i.e., able to integrate into their host’s genome to form a lysogen and subsequently transition to a lytic mode). We accomplished this by identifying the following three markers for temperate phages in the OVU representative sequences: (i) presence of phage integrase genes; (ii) presence of known prophage genes, according to the ACLAME (A CLAssification of Mobile genetic Elements) database; and (iii) nucleotide similarity to regions of bacterial genomes (29, 31, 32). We found that the majority of the phages were temperate and that the overall fraction of temperate phages remained consistent throughout the healthy, adenomatous, and cancerous stages (Fig. 3). These findings were consistent with previous reports suggesting that the gut virome is primarily composed of temperate phages (13, 18, 31, 33).

### Community context of influential phages.

**FIG 4:**
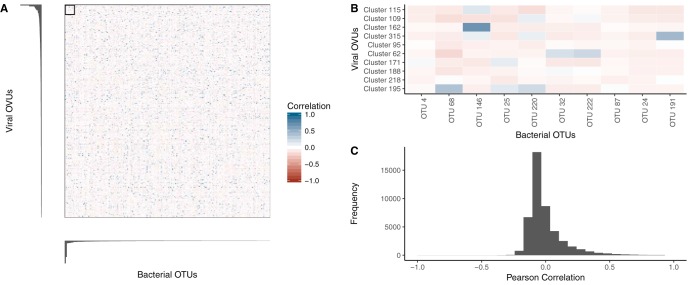
Relative-abundance correlations between bacterial OTUs and virome OVUs. (A) Pearson correlation coefficient values from comparisons between all bacterial OTUs (*x* axis) and viral OVUs (*y* axis), with blue representing positive correlations and red representing negative correlations. Bar plots indicate the levels of viral (left) and bacterial (bottom) operational unit importance in their colorectal cancer classification models, such that the most important units are shown in the top left corner. (B) Magnification of the boxed region in panel A, highlighting the correlation between the most important bacterial OTUs and virome OVUs. The most important operational units are shown in the top left corner of the heat map, and the correlation scale is the same as in panel A. (C) Histogram quantifying the frequencies of Pearson correlation coefficients between all bacterial OTUs and virome OVUs.

Because the link between colorectal cancer and the virome was driven by bacteriophages (rather than by nonbacterial viruses), we tested the potential hypothesis that the virome signal was a mere reflection of the bacterial signal and was thus highly correlated with the bacterial signal. If this hypothesis were true, we would expect a correlation between the relative abundances of influential bacterial OTUs and virome OVUs. Instead, we observed a strikingly low correlation between bacterial and viral relative abundances (Fig. 4A and C). Overall, there was an absence of correlation between the most influential OVUs and bacterial OTUs (Fig. 4B). This evidence supported our null hypothesis that the influential viral OVUs did not primarily represent reflections of the presence of influential bacteria.

**FIG 5:**
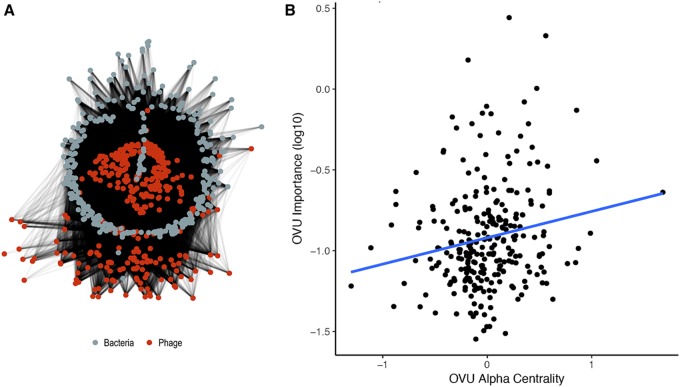
Community network analysis utilizing predicted interactions between bacterial and phage operational genomic units. (A) Visualization of the community network for our colorectal cancer cohort. (B) Scatter plot illustrating the correlation between importance (mean decrease in accuracy) and the degree of centrality for each OVU. A linear regression line was fitted to illustrate the correlations (blue) found to be statistically significantly and weakly correlated (*P* value = 0.00409, *R* = 0.176).

Given these findings, we posited that the most influential phages were acting by infecting a wide range of bacteria in the overall community instead of just the influential bacteria. In other words, we hypothesized that the influential bacteriophages were community hubs (i.e., central members) within the bacterium and phage interactive network. We investigated the potential host ranges of all phage OVUs using a previously developed random forest model that relies on sequence features to predict which phages infect which bacteria in the community (Fig. 5A) (34). The predicted interactions were then used to identify phage community hubs. We calculated the alpha centrality (i.e., the measure of importance in the ecological network) of the connection of the OVU of each phage to the rest of the network. The phages with high centrality values were defined as community hubs. Next, the centrality of each OVU was compared to its importance in the colorectal cancer classification model. Phage OVU centrality was significantly and positively correlated with importance to the disease model (*P* value = 0.004, *R* = 0.176), suggesting that the phages that were important in driving colorectal cancer also were more likely to be community hubs (Fig. 5B). Together, these findings supported our hypothesis that the influential phages were hubs within their microbial communities and had broad host ranges.

## DISCUSSION

Because of their propensity for mutagenesis and capacity for modulating their host functionality, many human viruses are oncogenic (1–4). Some bacteria also have oncogenic properties, suggesting that bacteriophages, representing a component of the human virome in addition to human-specific viruses, may play an indirect role in promoting carcinogenesis by influencing bacterial community composition and dynamics (8–10). Despite their carcinogenic potential and the strong association between bacteria and colorectal cancer, a link between virus colorectal communities and colorectal cancer has yet to be evaluated. Here we show that, like colonic bacterial communities, the colon virome was altered in patients with colorectal cancer relative to those with healthy colons. Our findings support a working hypothesis for oncogenesis by phage-modulated bacterial community composition.

**FIG 6:**
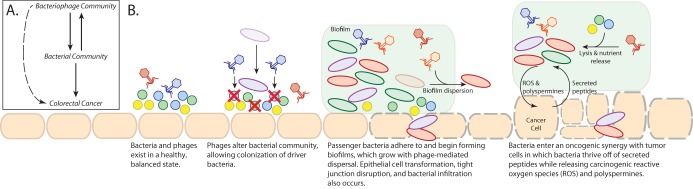
Final working hypothesis from this study. These panels summarize our thoughts on our results and represent interesting future directions that we predict will build on the presented work. (A) Basic model illustrating the connections between the virome, bacterial communities, and colorectal cancer. (B) Working hypothesis of how the bacteriophage community is associated with colorectal cancer and the associated bacterial community. ROS, reactive oxygen species.

On the basis of our findings, we have developed a conceptual model to be tested in our future studies aimed at elucidating the role that the colonic virome plays in colorectal cancer (Fig. 6A). We found that basic diversity metrics of alpha diversity (richness and Shannon diversity) and beta diversity (Bray-Curtis dissimilarity) were insufficient for identifying virome community differences between healthy and cancerous states. By implementing a machine learning approach (random forest classification) to leverage inherent, complex patterns not detected by diversity measures, we were able to detect strong associations between the colon virus community composition and colorectal cancer. The double-stranded DNA (dsDNA) virome of colorectal cancer was composed primarily of bacteriophages. These phage communities were not exclusively predators of the most influential bacteria, as demonstrated by the lack of correlation between the abundances of the bacterial and phage populations. Instead, we identified influential phages as being community hubs, suggesting that phages influence cancer by altering the greater bacterial community instead of directly modulating the influential bacteria. Our previous work has shown that modifying colon bacterial communities alters colorectal cancer progression and tumor burden in mice (10, 20). This provides a precedent for the idea of phages indirectly influencing colorectal cancer progression by altering the bacterial community composition. Overall, our data support a model in which the bacteriophage community modulates the bacterial community and, through those interactions, indirectly influences the bacteria driving colorectal cancer progression (Fig. 6A). Although our evidence suggested that phages indirectly influenced colorectal cancer development, we were not able to rule out a possible role of phages in direct interactions with the human host (35, 36).

In addition to modeling the potential connections between virus communities, bacterial communities, and colorectal cancer, we also used our data and existing knowledge of phage biology to develop a working hypothesis regarding the mechanisms by which this may occur. This was done by incorporating our findings into a current model for colorectal cancer development (Fig. 6B) (22), although it is important that there are also many other alternative hypotheses by which the system could be operating. We hypothesize that the process begins with broadly infectious phages in the colon lysing, and thereby disrupting, the existing bacterial communities. This shift opens novel niche space that enables opportunistic bacteria (such as Fusobacterium nucleatum) to colonize. Once the initial influential founder bacteria establish themselves in the epithelium, secondary opportunistic bacteria are able to adhere to the founders, colonize, and establish a biofilm. Phages may play a role in biofilm dispersal and growth by lysing bacteria within the biofilm, a process important for effective biofilm growth (37). The oncogenic bacteria may then be able to transform the epithelial cells and disrupt tight junctions to infiltrate the epithelium, thereby initiating an inflammatory immune response. As the adenomatous polyps developed and progressed toward carcinogenesis, we observed a shift in the phages and bacteria whose relative abundances were most influential. As the bacteria enter their state of oncogenic synergy with the epithelium, we conjecture that the phages continue mediating biofilm dispersal. This process would thereby support the colonized oncogenic bacteria by lysing competing cells and releasing nutrients to other bacteria in the form of cellular lysates. In addition to highlighting the likely mechanisms by which the colorectal cancer virome is interacting with the bacterial communities, this model will guide future research investigations of the role that the virome plays in colorectal cancer.

Our working hypothesis represents a conceptualization of areas for the future work that will be required for characterization of the colorectal cancer microbiome at the functional, mechanistic level. There are many different ways in which this system may operate, and our working hypothesis represents one of those ways. For example, it is possible that the bacterial communities cause a change in the virome instead of the virome altering the bacterial communities. To better understand this system, future studies will include larger-cohort human studies, further *in vitro* and *in vivo* mechanistic experimentation, and attempts at community studies using absolute abundance values instead of relative abundance, which would allow more-accurate community dynamic modeling. Overall, this report provides a conceptual foundation to direct future characterization of the colorectal cancer microbiome at the functional, mechanistic level.

In addition to the diagnostic ramifications of understanding the colorectal cancer microbiome, our findings suggest that viruses, while understudied and currently underappreciated in the human microbiome, are likely important contributors to human disease. Studies of viral community dynamics have the potential to provide an abundance of information to supplement data from bacterial communities. Evidence has suggested that the virome is a component that is crucial to the microbiome and that bacteriophages are important players. Bacteriophage and bacterial communities cannot maintain stability and coevolution without one another (6, 38). Not only is the human virome an important element to consider in human health and disease (12–18), but our findings support the concept that it is likely to have a significant impact on cancer etiology and progression.

## MATERIALS AND METHODS

### Study design and patient sampling.

This study was approved by the University of Michigan Institutional Review Board, and all subjects provided informed consent. The design of and sampling method used for this sample set have been reported previously (8). Briefly, whole evacuated stool was collected from patients who were 18 years of age or older, able to provide informed consent, had had colonoscopy and histologically confirmed colonic disease status, had not had surgery, had not had chemotherapy or radiation, and were free of known comorbidities, including HIV, chronic viral hepatitis, hereditary nonpolyposis colorectal cancer (HNPCC), familial adenomatous polyposis (FAP), and inflammatory bowel disease. Healthy subjects entered the clinic for the study and did not present as a result of comorbities. Samples were collected from four geographic locations: Toronto (Ontario, Canada), Boston (MA, USA), Houston (TX, USA), and Ann Arbor (MI, USA). Ninety patients were recruited to the study, thirty of whom were designated healthy, thirty with detected adenomas, and thirty with detected carcinomas.

### 16S rRNA gene sequence data acquisition and processing.

The 16S rRNA gene sequences associated with this study were previously reported (8). Sequence (fastq) and metadata files were downloaded from the following site: http://www.mothur.org/MicrobiomeBiomarkerCRC.

The 16S rRNA gene sequences were analyzed as described previously, relying on the mothur software package (v1.37.0) (39, 40). Briefly, the sequences were dereplicated, aligned to the SILVA database (41), screened for chimeras using UCHIME (42), and binned into operational taxonomic units (OTUs) using a 97% similarity threshold. Abundances were normalized for uneven sequencing depth by randomly subsampling to 10,000 sequences, as previously reported (23).

### Whole-metagenomic-library preparation and sequencing.

DNA was extracted from stool samples using a PowerSoil HTP 96-well soil DNA isolation kit (Mo Bio Laboratories) and an EPMotion 5075 pipetting system. Purified DNA was used to prepare a shotgun sequencing library using an Illumina Nextera XT library preparation kit according to the standard kit protocol, including 12 cycles of limited-cycle PCR. The tagmentation time was increased from 5 min to 10 min to improve the DNA fragment length distribution. The library was sequenced using one lane of an Illumina HiSeq 4000 platform and yielded 125-bp paired-end reads.

### Virus metagenomic library preparation and sequencing.

Genomic DNA was extracted from purified virus-like particles (VLPs) from stool samples, using a modified version of a previously published protocol (29, 31, 43, 44). Briefly, an aliquot of stool (∼0.1 g) was resuspended in SM buffer (Crystalgen; catalog no. 221-179) and subjected to vortex mixing to facilitate resuspension. The resuspended stool was centrifuged to remove major particulate debris and then filtered through a 0.22-µm-pore-size filter to remove smaller contaminants. The filtered supernatant was treated with chloroform for 10 min with gentle shaking to lyse contaminating cells, including bacterial cells, human cells, fungal cells, etc. The exposed genomic DNA from the lysed cells was degraded by treating the samples with 5 U of DNase for 1 h at 37°C. DNase was deactivated by incubating the sample at 75°C for 10 min. The DNA was extracted from the purified virus-like particles (VLPs) using a Wizard PCR purification preparation kit (Promega). Disease classes were staggered across purification runs to prevent run variation from becoming a confounding factor. As for the whole-community metagenomes, purified DNA was used to prepare a shotgun sequencing library using an Illumina Nextera XT preparation kit according to the standard kit protocol. The tagmentation time was increased from 5 min to 10 min to improve the DNA fragment length distribution. The PCR cycle number was increased from 12 to 18 cycles to address the low biomass of the samples as described previously (29). The library was sequenced using one lane of the Illumina HiSeq 4000 platform and yielded 125-bp paired-end reads.

### Metagenome quality control.

The viral and whole-community metagenomic sample sets were subjected to the same quality control procedures. The sequences were obtained as demultiplexed fastq files and subjected to 5′ and 3′ adapter trimming using the CutAdapt program (v1.9.1) with an error rate of 0.1 and an overlap of 10 (45). The FastX toolkit (v0.0.14) was used to quality trim the reads to a minimum length of 75 bp and a minimum quality score of 30 (46). Reads mapping to the human genome were removed using the DeconSeq algorithm (v0.4.3) and default parameters (47).

### Contig assembly and abundance.

Contigs were assembled using paired-end read files that were purged of sequences without a corresponding pair (e.g., one read was removed due to low quality). The Megahit program (v1.0.6) was used to assemble contigs for each sample using a minimum contig length of 1,000 bp and iterating assemblies from 21-mers to 101-mers by 20 (48). Contigs from the virus and whole-metagenomic sample sets were concatenated within their respective groups. The abundance of the contigs within each sample was calculated by realigning sequences to the concatenated contig files using the bowtie2 global aligner (v2.2.1) with a 25-bp seed length and an allowance of one mismatch (49). Abundance was corrected for contig reference length and for the number of contigs included in each operational genomic unit. Abundance was also corrected for uneven sampling depth by randomly subsampling virome and whole metagenomes to depths of 1,000,000 and 500,000 reads, respectively, and by removing samples with fewer total reads than the threshold. Thresholds were set for maximizing sequence information while minimizing numbers of lost samples.

### Operational genomic unit classification.

In much the same manner that operational taxonomic units (OTUs) are used as an operational definition of similar 16S rRNA gene sequences, we defined closely related bacterial contig sequences as operational genomic units (OGUs) and virus contigs as operational viral units (OVUs) in the absence of taxonomic identity. OGUs and OVUs were defined with the CONCOCT algorithm (v0.4.0)m which bins related contigs by similar tetramer and coabundance profiles within samples by the use of a variation-based Bayesian approach (50). CONCOCT was used with length thresholds of 1,000 bp for virus contigs and 2,000 bp for bacteria.

### Diversity.

Alpha and beta diversity were calculated using the operational viral unit abundance profiles for each sample. Sequences were rarefied to 100,000 sequences. Samples with less than the cutoff value were removed from the analysis. Alpha diversity was calculated using the Shannon diversity and richness metrics. Beta diversity was calculated using the Bray-Curtis metric (mean of 25 random subsampling iterations), and the statistical significance of results of comparisons between the disease state clusters was assessed using analysis of similarity (ANOSIM) with a *post hoc* multivariate Tukey test. All diversity calculations were performed in R using the Vegan package (51).

### Classification modeling.

Classification modeling was performed in R using the Caret package (52). OTU, OVU, and OGU abundance data were preprocessed by removing features (OTUs, OVUs, and OGUs) that were present in fewer than thirty of the samples. This method both served as an effective feature reduction technique and made the calculations computationally feasible. The binary random forest model was trained using the area under the receiver operating characteristic curve (AUC), and the three-class random forest model was trained using the mean AUC. Both were validated using 5-fold nested cross-validation to prevent overfitting of the tuning parameters. Each training set step was repeated five times, and the model was tuned for mtry values. For consistency and accurate comparisons between feature groups (e.g., bacteria and viruses), the sample model parameters were used for each group. The maximum AUC during training was recorded across 20 iterations of each group model to test the significance of the differences between feature set performances. Statistical significance was evaluated using a Wilcoxon test for comparisons between two categories or a pairwise Wilcoxon test with Bonferroni-corrected *P* values for comparisons among three or more categories.

### Taxonomic identification of operational genomic units.

Operational viral units (OVUs) were taxonomically identified using a reference database consisting of all bacteriophage and eukaryotic virus genomes present in the European Nucleotide Archives. The longest contiguous sequence in each operational genomic unit was used as a representative sequence for classification, as described previously (53). Each representative sequence was aligned to the reference genome database using the tblastx alignment algorithm (v2.2.27) and a strict similarity threshold (E value < 1e−25) (54). The annotation results were interpreted as “phage,” “eukaryotic virus,” or “unknown.” As an additional quality control step, these OVUs were also aligned to the bacterial reference genome set from the European Nucleotide Archives using the blastn algorithm (E value < 1e−25), and OVUs with similarity to bacterial genomes and not viral genomes were removed from analysis.

### Ecological network analysis and correlations.

The ecological network of the bacterial and phage operational genomic units was constructed and analyzed as previously described (34). Briefly, a random forest model was used to predict interactions between bacterial and phage genomic units, and those interactions were recorded in a graph database using *neo4j* graph databasing software (v2.3.1). The degree of phage centrality was quantified using the alpha centrality metric in the igraph CRAN package. A Spearman correlation analysis was performed for comparisons between model importance and phage centrality scores.

### Phage replication style identification.

The phage OVU replication mode was predicted using methods described previously (29, 31, 32). Briefly, we identified temperate OVUs as representative contigs containing at least one of the following three genomic markers: (i) phage integrase genes, (ii) prophage genes from the ACLAME database, and (iii) genomic similarity to bacterial reference genomes. Integrase genes were identified in phage OVU representative contigs by aligning the contigs to a reference database of all known phage integrase genes from the Uniprot database (Uniprot search term: "organism:phage gene:int NOT putative"). Prophage genes were identified in the same way, using the ACLAME set of reference prophage genes. In both cases, the blastx algorithm was used with an E value threshold of 10e−5. Representative contigs that had a high genomic similarity to bacterial genomes were also identified as potential lysogenic phages. To accomplish this, representative phage contigs were aligned to the European Nucleotide Archive bacterial genome reference set using the blastn algorithm (E value < 10e−25).

### Data availability.

All study sequences are available on the NCBI Sequence Read Archive under BioProject identifier (ID) PRJNA389927. All associated source codes are available at the following GitHub repository: https://github.com/SchlossLab/Hannigan_CRCVirome_mBio_2018.

## References

[1] Feng H, Shuda M, Chang Y, Moore PS 2008 Clonal integration of a polyomavirus in human Merkel cell carcinoma. Science 319:1096–1100. doi:10.1126/science.1152586. 18202256 PMC2740911

[2] Shuda M, Kwun HJ, Feng H, Chang Y, Moore PS 2011 Human Merkel cell polyomavirus small T antigen is an oncoprotein targeting the 4E-BP1 translation regulator. J Clin Invest 121:3623–3634. doi:10.1172/JCI46323. 21841310 PMC3163959

[3] Schiller JT, Castellsagué X, Garland SM 2012 A review of clinical trials of human papillomavirus prophylactic vaccines. Vaccine 30:F123–F138. doi:10.1016/j.vaccine.2012.04.108. 23199956 PMC4636904

[4] Chang Y, Cesarman E, Pessin MS, Lee F, Culpepper J, Knowles DM, Moore PS 1994 Identification of herpesvirus-like DNA sequences in AIDS-associated Kaposi’s sarcoma. Science 266:1865–1869. doi:10.1126/science.7997879. 7997879

[5] Harcombe WR, Bull JJ 2005 Impact of phages on two-species bacterial communities. Appl Environ Microbiol 71:5254–5259. doi:10.1128/AEM.71.9.5254-5259.2005. 16151111 PMC1214695

[6] Rodriguez-Valera F, Martin-Cuadrado A-B, Rodriguez-Brito B, Pašić L, Thingstad TF, Rohwer F, Mira A 2009 Explaining microbial population genomics through phage predation. Nat Rev Microbiol 7:828–836. doi:10.1038/nrmicro2235. 19834481

[7] Cortez MH, Weitz JS 2014 Coevolution can reverse predator-prey cycles. Proc Natl Acad Sci U S A 111:7486–7491. doi:10.1073/pnas.1317693111. 24799689 PMC4034221

[8] Zackular JP, Rogers MAM, Ruffin MT, Schloss PD 2014 The human gut microbiome as a screening tool for colorectal cancer. Cancer Prevention Res 7:1112–1121. doi:10.1158/1940-6207.CAPR-14-0129. PMC4221363 25104642

[9] Garrett WS 2015 Cancer and the microbiota. Science 348:80–86. doi:10.1126/science.aaa4972. 25838377 PMC5535753

[10] Baxter NT, Zackular JP, Chen GY, Schloss PD 2014 Structure of the gut microbiome following colonization with human feces determines colonic tumor burden. Microbiome 2:20. doi:10.1186/2049-2618-2-20. 24967088 PMC4070349

[11] Arthur JC, Perez-Chanona E, Mühlbauer M, Tomkovich S, Uronis JM, Fan T-J, Campbell BJ, Abujamel T, Dogan B, Rogers AB, Rhodes JM, Stintzi A, Simpson KW, Hansen JJ, Keku TO, Fodor AA, Jobin C 2012 Intestinal inflammation targets cancer-inducing activity of the microbiota. Science 338:120–123. doi:10.1126/science.1224820. 22903521 PMC3645302

[12] Ly M, Abeles SR, Boehm TK, Robles-Sikisaka R, Naidu M, Santiago-Rodriguez T, Pride DT 2014 Altered oral viral ecology in association with periodontal disease. mBio 5:e01133-14. doi:10.1128/mBio.01133-14. 24846382 PMC4030452

[13] Monaco CL, Gootenberg DB, Zhao G, Handley SA, Ghebremichael MS, Lim ES, Lankowski A, Baldridge MT, Wilen CB, Flagg M, Norman JM, Keller BC, Luévano JM, Wang D, Boum Y, Martin JN, Hunt PW, Bangsberg DR, Siedner MJ, Kwon DS, Virgin HW 2016 Altered virome and bacterial microbiome in human immunodeficiency virus-associated acquired immunodeficiency syndrome. Cell Host Microbe 19:311–322. doi:10.1016/j.chom.2016.02.011. 26962942 PMC4821831

[14] Willner D, Furlan M, Haynes M, Schmieder R, Angly FE, Silva J, Tammadoni S, Nosrat B, Conrad D, Rohwer F 2009 Metagenomic analysis of respiratory tract DNA viral communities in cystic fibrosis and non-cystic fibrosis individuals. PLoS One 4:e7370. doi:10.1371/journal.pone.0007370. 19816605 PMC2756586

[15] Abeles SR, Ly M, Santiago-Rodriguez TM, Pride DT 2015 Effects of long term antibiotic therapy on human oral and fecal viromes. PLoS One 10:e0134941. doi:10.1371/journal.pone.0134941. 26309137 PMC4550281

[16] Modi SR, Lee HH, Spina CS, Collins JJ 2013 Antibiotic treatment expands the resistance reservoir and ecological network of the phage metagenome. Nature 499:219–222. doi:10.1038/nature12212. 23748443 PMC3710538

[17] Santiago-Rodriguez TM, Ly M, Bonilla N, Pride DT 2015 The human urine virome in association with urinary tract infections. Front Microbiol 6:14. doi:10.3389/fmicb.2015.00014. 25667584 PMC4304238

[18] Norman JM, Handley SA, Baldridge MT, Droit L, Liu CY, Keller BC, Kambal A, Monaco CL, Zhao G, Fleshner P, Stappenbeck TS, McGovern DPB, Keshavarzian A, Mutlu EA, Sauk J, Gevers D, Xavier RJ, Wang D, Parkes M, Virgin HW 2015 Disease-specific alterations in the enteric virome in inflammatory bowel disease. Cell 160:447–460. doi:10.1016/j.cell.2015.01.002. 25619688 PMC4312520

[19] Siegel R, Desantis C, Jemal A 2014 Colorectal cancer statistics, 2014. CA Cancer J Clin 64:104–117. doi:10.3322/caac.21220. 24639052

[20] Zackular JP, Baxter NT, Chen GY, Schloss PD 2016 Manipulation of the gut microbiota reveals role in colon tumorigenesis. mSphere 1:e00001-15. doi:10.1128/mSphere.00001-15. 27303681 PMC4863627

[21] Dejea CM, Wick EC, Hechenbleikner EM, White JR, Mark Welch JL, Rossetti BJ, Peterson SN, Snesrud EC, Borisy GG, Lazarev M, Stein E, Vadivelu J, Roslani AC, Malik AA, Wanyiri JW, Goh KL, Thevambiga I, Fu K, Wan F, Llosa N, Housseau F, Romans K, Wu X, McAllister FM, Wu S, Vogelstein B, Kinzler KW, Pardoll DM, Sears CL 2014 Microbiota organization is a distinct feature of proximal colorectal cancers. Proc Natl Acad Sci U S A 111:18321–18326. doi:10.1073/pnas.1406199111. 25489084 PMC4280621

[22] Flynn KJ, Baxter NT, Schloss PD 2016 Metabolic and community synergy of oral bacteria in colorectal cancer. mSphere 1:e00102-16. doi:10.1128/mSphere.00102-16. 27303740 PMC4888883

[23] Baxter NT, Ruffin MT, Rogers MAM, Schloss PD 2016 Microbiota-based model improves the sensitivity of fecal immunochemical test for detecting colonic lesions. Genome Medicine 8:37. doi:10.1186/s13073-016-0290-3. 27056827 PMC4823848

[24] Zeller G, Tap J, Voigt AY, Sunagawa S, Kultima JR, Costea PI, Amiot A, Böhm J, Brunetti F, Habermann N, Hercog R, Koch M, Luciani A, Mende DR, Schneider MA, Schrotz-King P, Tournigand C, Tran Van Nhieu J, Yamada T, Zimmermann J, Benes V, Kloor M, Ulrich CM, von Knebel Doeberitz M, Sobhani I, Bork P 2014 Potential of fecal microbiota for early-stage detection of colorectal cancer. Mol Syst Biol 10:766–766. doi:10.15252/msb.20145645. 25432777 PMC4299606

[25] Fearon ER 2011 Molecular genetics of colorectal cancer. Annu Rev Pathol 6:479–507. doi:10.1146/annurev-pathol-011110-130235. 21090969

[26] Levin B, Lieberman DA, McFarland B, Smith RA, Brooks D, Andrews KS, Dash C, Giardiello FM, Glick S, Levin TR, Pickhardt P, Rex DK, Thorson A, Winawer SJ; American Cancer Society Colorectal Cancer Advisory Group, US Multi-Society Task Force, American College of Radiology Colon Cancer Committee. 2008 Screening and surveillance for the early detection of colorectal cancer and adenomatous polyps, 2008: a joint guideline from the American Cancer Society, the US Multi-Society Task Force on Colorectal Cancer, and the American College of Radiology. CA Cancer J Clin 58:130–160. doi:10.3322/CA.2007.0018. 18322143

[27] Zauber AG 2015 The impact of screening on colorectal cancer mortality and incidence: has it really made a difference? Dig Dis Sci 60:681–691. doi:10.1007/s10620-015-3600-5. 25740556 PMC4412262

[28] Pedulla ML, Ford ME, Houtz JM, Karthikeyan T, Wadsworth C, Lewis JA, Jacobs-Sera D, Falbo J, Gross J, Pannunzio NR, Brucker W, Kumar V, Kandasamy J, Keenan L, Bardarov S, Kriakov J, Lawrence JG, Jacobs WR, Hendrix RW, Hatfull GF 2003 Origins of highly mosaic mycobacteriophage genomes. Cell 113:171–182. doi:10.1016/S0092-8674(03)00233-2. 12705866

[29] Hannigan GD, Meisel JS, Tyldsley AS, Zheng Q, Hodkinson BP, SanMiguel AJ, Minot S, Bushman FD, Grice EA 2015 The human skin double-stranded DNA virome: topographical and temporal diversity, genetic enrichment, and dynamic associations with the host microbiome. mBio 6:e01578-15. doi:10.1128/mBio.01578-15. 26489866 PMC4620475

[30] Brum JR, Ignacio-Espinoza JC, Roux S, Doulcier G, Acinas SG, Alberti A, Chaffron S, Cruaud C, Vargas C, de Gasol JM, Gorsky G, Gregory AC, Guidi L, Hingamp P, Iudicone D, Not F, Ogata H, Pesant S, Poulos BT, Schwenck SM, Speich S, Dimier C, Kandels-Lewis S, Picheral M, Searson S, Tara Oceans Coordinators, Bork P, Bowler C, Sunagawa S, Wincker P, Karsenti E, Sullivan MB 2015 Ocean plankton. Patterns and ecological drivers of ocean viral communities. Science 348:1261498–1261498. doi:10.1126/science.1261498. 25999515

[31] Minot S, Sinha R, Chen J, Li H, Keilbaugh SA, Wu GD, Lewis JD, Bushman FD 2011 The human gut virome: inter-individual variation and dynamic response to diet. Genome Res 21:1616–1625. doi:10.1101/gr.122705.111. 21880779 PMC3202279

[32] Hannigan GD, Zheng Q, Meisel JS, Minot SS, Bushman FD, Grice EA 2017 Evolutionary and functional implications of hypervariable loci within the skin virome. PeerJ 5:e2959. doi:10.7717/peerj.2959. 28194314 PMC5299996

[33] Reyes A, Haynes M, Hanson N, Angly FE, Heath AC, Rohwer F, Gordon JI 2010 Viruses in the faecal microbiota of monozygotic twins and their mothers. Nature 466:334–338. doi:10.1038/nature09199. 20631792 PMC2919852

[34] Hannigan GD, Duhaime MB, Koutra D, Schloss PD 2018 Biogeography and environmental conditions shape bacteriophage-bacteria networks across the human microbiome. PLoS Comput Biol 14:e1006099. doi:10.1371/journal.pcbi.1006099. 29668682 PMC5927471

[35] Lengeling A, Mahajan A, Gally DL 2013 Bacteriophages as Pathogens and Immune Modulators? mBio 4:e00868-13. doi:10.1128/mBio.00868-13. 24222490 PMC3870245

[36] Górski A, Międzybrodzki R, Borysowski J, Dąbrowska K, Wierzbicki P, Ohams M, Korczak-Kowalska G, Olszowska-Zaremba N, Łusiak-Szelachowska M, Kłak M, Jończyk E, Kaniuga E, Gołaś A, Purchla S, Weber-Dąbrowska B, Letkiewicz S, Fortuna W, Szufnarowski K, Pawełczyk Z, Rogóż P, Kłosowska D 2012 Phage as a modulator of immune responses: practical implications for phage therapy. Adv Virus Res 83:41–71. doi:10.1016/B978-0-12-394438-2.00002-5. 22748808

[37] Rossmann FS, Racek T, Wobser D, Puchalka J, Rabener EM, Reiger M, Hendrickx APA, Diederich A-K, Jung K, Klein C, Huebner J 2015 Phage-mediated dispersal of biofilm and distribution of bacterial virulence genes is induced by quorum sensing. PLoS Pathog 11:e1004653. doi:10.1371/journal.ppat.1004653. 25706310 PMC4338201

[38] Brockhurst MA, Koskella B 2013 Experimental coevolution of species interactions. Trends Ecol Evol 28:367–375. doi:10.1016/j.tree.2013.02.009. 23523051

[39] Kozich JJ, Westcott SL, Baxter NT, Highlander SK, Schloss PD 2013 Development of a dual-index sequencing strategy and curation pipeline for analyzing amplicon sequence data on the MiSeq Illumina sequencing platform. Appl Environ Microbiol 79:5112–5120. doi:10.1128/AEM.01043-13. 23793624 PMC3753973

[40] Schloss PD, Westcott SL, Ryabin T, Hall JR, Hartmann M, Hollister EB, Lesniewski RA, Oakley BB, Parks DH, Robinson CJ, Sahl JW, Stres B, Thallinger GG, Van Horn DJ, Weber CF 2009 Introducing mothur: open-source, platform-independent, community-supported software for describing and comparing microbial communities. Appl Environ Microbiol 75:7537–7541. doi:10.1128/AEM.01541-09. 19801464 PMC2786419

[41] Pruesse E, Quast C, Knittel K, Fuchs BM, Ludwig W, Peplies J, Glöckner FO 2007 SILVA: a comprehensive online resource for quality checked and aligned ribosomal RNA sequence data compatible with ARB. Nucleic Acids Res 35:7188–7196. doi:10.1093/nar/gkm864. 17947321 PMC2175337

[42] Edgar RC, Haas BJ, Clemente JC, Quince C, Knight R 2011 UCHIME improves sensitivity and speed of chimera detection. Bioinformatics 27:2194–2200. doi:10.1093/bioinformatics/btr381. 21700674 PMC3150044

[43] Thurber RV, Haynes M, Breitbart M, Wegley L, Rohwer F 2009 Laboratory procedures to generate viral metagenomes. Nat Protoc 4:470–483. doi:10.1038/nprot.2009.10. 19300441

[44] Kleiner M, Hooper LV, Duerkop BA 2015 Evaluation of methods to purify virus-like particles for metagenomic sequencing of intestinal viromes. BMC Genomics 16:7. doi:10.1186/s12864-014-1207-4. 25608871 PMC4308010

[45] Martin M 2011 Cutadapt removes adapter sequences from high-throughput sequencing reads. EMBnet J 17:10. doi:10.14806/ej.17.1.200.

[46] Hannon GJ 2010 FASTX-Toolkit GNU Affero General Public License. https://www.gnu.org/licenses/agpl-3.0-standalone.html.

[47] Schmieder R, Edwards R 2011 Fast identification and removal of sequence contamination from genomic and metagenomic datasets. PLoS One 6:e17288. doi:10.1371/journal.pone.0017288. 21408061 PMC3052304

[48] Li D, Luo R, Liu C-M, Leung C-M, Ting H-F, Sadakane K, Yamashita H, Lam T-W 2016 MEGAHIT v1.0: a fast and scalable metagenome assembler driven by advanced methodologies and community practices. Methods 102:3–11. doi:10.1016/j.ymeth.2016.02.020. 27012178

[49] Langmead B, Salzberg SL 2012 Fast gapped-read alignment with Bowtie 2. Nat Methods 9:357–359. doi:10.1038/nmeth.1923. 22388286 PMC3322381

[50] Alneberg J, Bjarnason BS, Bruijn I, Schirmer M, Quick J, Ijaz UZ, Lahti L, Loman NJ, Andersson AF, Quince C 2014 Binning metagenomic contigs by coverage and composition. Nat Methods 11:1144–1146. doi:10.1038/nmeth.3103. 25218180

[51] Oksanen J, Blanchet FG, Friendly M, Kindt R, Legendre P, McGlinn D, Minchin PR, OHara RB, Simpson GL, Solymos P, Stevens MHH, Szoecs E, Wagner H 2018 vegan: community ecology package. http://vegan.r-forge.r-project.org/.

[52] Kuhn M 2016 caret: classification and regression training. https://www.researchgate.net/publication/292021862_Caret_Classification_and_regression_training.

[53] Guidi L, Chaffron S, Bittner L, Eveillard D, Larhlimi A, Roux S, Darzi Y, Audic S, Berline L, Brum JR, Coelho LP, Espinoza JCI, Malviya S, Sunagawa S, Dimier C, Kandels-Lewis S, Picheral M, Poulain J, Searson S, Tara Oceans Consortium Coordinators, Stemmann L, Not F, Hingamp P, Speich S, Follows M, Karp-Boss L, Boss E, Ogata H, Pesant S, Weissenbach J, Wincker P, Acinas SG, Bork P, de Vargas C, Iudicone D, Sullivan MB, Raes J, Karsenti E, Bowler C, Gorsky G 2016 Plankton networks driving carbon export in the oligotrophic ocean. Nature 532:465–470. doi:10.1038/nature16942. 26863193 PMC4851848

[54] Camacho C, Coulouris G, Avagyan V, Ma N, Papadopoulos J, Bealer K, Madden TL 2009 BLAST+: architecture and applications. BMC Bioinformatics 10:421. doi:10.1186/1471-2105-10-421. 20003500 PMC2803857

