# Supplementary figures and images for: Diagnostic Potential and Interactive Dynamics of the Colorectal Cancer Virome
Source: mBio. 2018 Nov 20;9(6):e02248-18. doi: 10.1128/mBio.02248-18 (PMC6247079; doi:10.1128/mBio.02248-18)

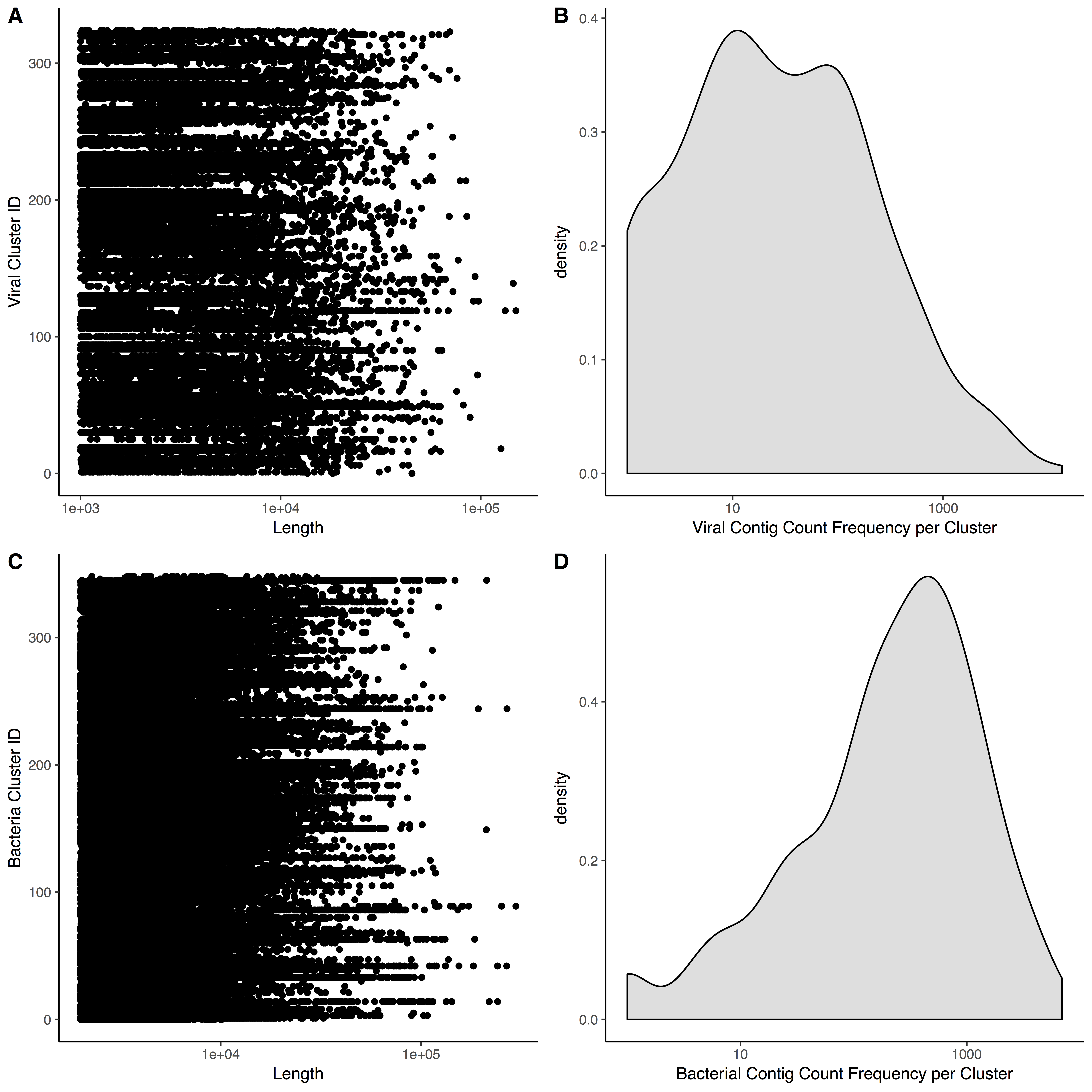

Supplement: FIG S3 [file mbo006184183sf3.tif]
